# Low-Intensity Pulsed Ultrasound Induces Angiogenesis and Ameliorates Left Ventricular Dysfunction in a Porcine Model of Chronic Myocardial Ischemia

**DOI:** 10.1371/journal.pone.0104863

**Published:** 2014-08-11

**Authors:** Kenichiro Hanawa, Kenta Ito, Kentaro Aizawa, Tomohiko Shindo, Kensuke Nishimiya, Yuhi Hasebe, Ryuji Tuburaya, Hideyuki Hasegawa, Satoshi Yasuda, Hiroshi Kanai, Hiroaki Shimokawa

**Affiliations:** 1 Department of Cardiovascular Medicine, Tohoku University Graduate School of Medicine, Sendai, Japan; 2 Division of Biomedical Measurements and Diagnostics, Graduate School of Biomedical Engineering, Tohoku University, Sendai, Japan; 3 Department of Electronic Engineering, Graduate School of Engineering, Tohoku University, Sendai, Japan; 4 National Cerebral and Cardiovascular Center, Osaka, Japan; University of Bristol, United Kingdom

## Abstract

**Background:**

Although a significant progress has been made in the management of ischemic heart disease (IHD), the number of severe IHD patients is increasing. Thus, it is crucial to develop new, non-invasive therapeutic strategies. In the present study, we aimed to develop low-intensity pulsed ultrasound (LIPUS) therapy for the treatment of IHD.

**Methods and Results:**

We first confirmed that in cultured human endothelial cells, LIPUS significantly up-regulated mRNA expression of vascular endothelial growth factor (VEGF) with a peak at 32-cycle (P<0.05). Then, we examined the in vivo effects of LIPUS in a porcine model of chronic myocardial ischemia with reduced left ventricular ejection fraction (LVEF) (n = 28). The heart was treated with either sham (n = 14) or LIPUS (32-cycle with 193 mW/cm^2^ for 20 min, n = 14) at 3 different short axis levels. Four weeks after the treatment, LVEF was significantly improved in the LIPUS group (46±4 to 57±5%, P<0.05) without any adverse effects, whereas it remained unchanged in the sham group (46±5 to 47±6%, P = 0.33). Capillary density in the ischemic region was significantly increased in the LIPUS group compared with the control group (1084±175 vs. 858±151/mm^2^, P<0.05). Regional myocardial blood flow was also significantly improved in the LIPUS group (0.78±0.2 to 1.39±0.4 ml/min/g, P<0.05), but not in the control group (0.84±0.3 to 0.97±0.4 ml/min/g). Western blot analysis showed that VEGF, eNOS and bFGF were all significantly up-regulated only in the LIPUS group.

**Conclusions:**

These results suggest that the LIPUS therapy is promising as a new, non-invasive therapy for IHD.

## Introduction

Ischemic heart disease (IHD) is one of the major causes of death in developed countries, and its morbidity is also increasing in developing countries [1]–[3]. Although recent advances in therapeutic strategies have reduced the mortality of patients with IHD [1], the number of severe IHD patients is increasing as the population is rapidly aging. Thus, non-invasive therapeutic strategies for severe IHD remain to be developed. We have previously demonstrated that low-energy extracorporeal cardiac shock wave (SW) therapy improves myocardial ischemia in a porcine model of chronic myocardial ischemia and patients with severe angina pectoris [4]–[8].

Ultrasound is a form of sound whose frequency is higher than the natural audible range for humans (>20 kHz) and ultrasonography has been widely used as diagnostic devices for several decades. In addition to diagnostic purposes, ultrasound is clinically used for therapeutic applications, including tumor ablation, thrombolysis, bone regeneration, and facilitated drug delivery [9]. Recently, therapeutic angiogenic effects of low-intensity ultrasound have been reported in endothelial cells, chick chorioallantoic membrane, and a rat model of hind limb ischemia [10]–[12]. In the present study, we thus examined whether low-intensity pulsed ultrasound (LIPUS) enhances endothelial regeneration in vitro and whether it ameliorates ischemia-induced myocardial dysfunction in a porcine model of chronic myocardial ischemia in vivo, and if so, what molecular mechanisms are involved. In addition, we also examined whether we can modify the current diagnostic ultrasound devices into the LIPUS therapy apparatus, in order to facilitate this LIPUS-mediated angiogenic therapy in the real world practice of IHD.

## Materials and Methods

All procedures were performed according to the protocols approved by the Institutional Committee for Use and Care of Laboratory Animals of Tohoku University (25MdA-36).

### Measurement of Acoustic Pressure of LIPUS

**Figure 1:**
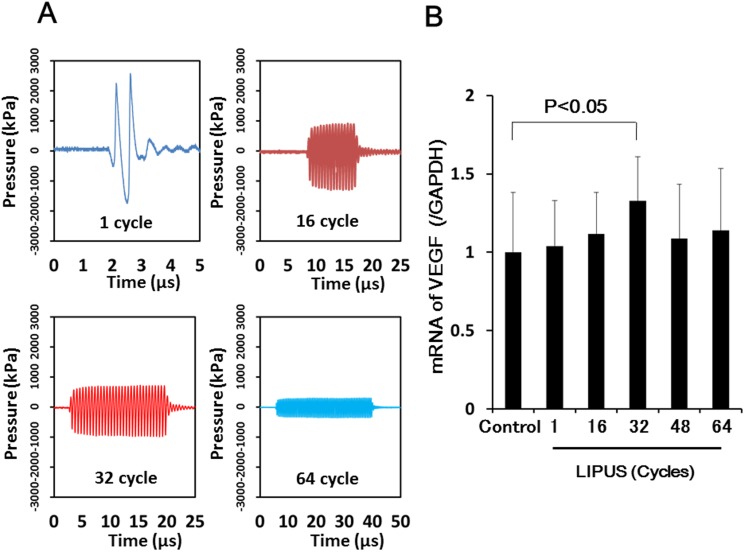
In vitro study. (**A**) Acoustic pressure at various cycle numbers. (**B**) LIPUS treatment up-regulated mRNA expression of VEGF in HUVECs in vitro with a maximum effect noted at 32-cycle. Results are expressed as mean ± SEM (n = 9–11 each).

We measured acoustic pressure of LIPUS under various conditions [frequency, 1.875 MHz; pulse repetition frequency, 7.10 kHz; number of cycles, 1, 16, 32, 48 and 64; voltage applied to each transducer element, 11.30∼88.18 volts (V); Ispta, 151∼193 mW/cm^2^] by using a needle hydrophone. The number of cycles of pulsed ultrasound means the number of acoustic waves per 1 pulse, while 1 cycle is used for diagnostic ultrasound devices. In the present study, however, we tested larger numbers of cycles from 16 to 64 (**Figure 1**). The voltage applied to each transducer element was controlled to keep the estimated spatial peak temporal average intensity (Ispta) of LIPUS below the upper limit of acoustic output standards (<720 mW/cm^2^) for diagnostic ultrasound devices (U.S. Food and Drug Administration’s Track 3 Limits) and to prevent the ultrasound probe from temperature rise.

### Effects of LIPUS on Human Umbilical Vein Endothelial Cells in Vitro

We purchased single-donor human umbilical vein endothelial cells (HUVEC) (Lonza, Basel, Switzerland) and cultured them in a complete endothelial medium (EGM-2 BulletKit, Lonza). HUVEC were subcultured and used at passages 3 to 5 and were maintained in EGM-2. For the LIPUS treatment, we used a diagnostic ultrasound device (Prosound α10; HITACHI Aloka Medical, Ltd., Mitaka, Japan) whose irradiation conditions could be modified by software modification. Twenty-four hours before the LIPUS treatment, HUVEC (1×10^5^) were re-suspended in a 2-ml tube with EGM (Lonza). The cells were exposed to LIPUS of various conditions [frequency, 1.875 MHz; pulse repetition frequency, 7.10 kHz; number of cycles, 1, 16, 32, 48 and 64 cycles; voltage applied to each transducer element, 11.30∼88.18 volts (V); Ispta, 151∼193 mW/cm^2^] for 20 minutes (n = 9–11 each). After irradiation, the cells were stored for 3 hours in the same medium before RNA extraction and for 20 minutes before protein extraction.

### Real-Time Polymerase Chain Reaction and Western Blot Analysis in vitro

Total RNA was extracted using the RNeasy Micro Kit (Qiagen, Hilden, Germany). Total RNA (600 ng) was reverse-transcribed using a QuantiTect Reverse Transcription Kit (Qiagen). Real-time polymerase chain reaction (RT-PCR) was performed using the Real-Time Detection System (Bio-Rad Laboratories, Hercules, CA). Sequences of the primers were (forward, reverse) 5′-GAGCCTTGCCTTGCTGCTCTA-3′, 5′-CACCAGGGTCTCGATTGGATG-3′ for vascular endothelial growth factor (VEGF), 5′- GTGTGCTAACCGTTACCTGGCTATG-3′, 5′- CCAGTTCGTTTCAGTGCCACA-3′ for basic fibroblast growth factor (bFGF), 5′- TTCTGACTGCACAAACCAGCTTC-3′, 5′-TTTGACACCACACACAGCTTCAC-3′ for Flk-1, and 5′-GCACCGTCAAGGCTGAGAAC-3′, 5′-TGGTGAAGACGCCAGTGGA-3′ for β-actin, all of which were designed by the Perfect Real Time Support System (Takara Bio Inc., Otsu, Japan). The β-actin was used as an internal control and SYBR Premix Ex Taq I and II (Takara Bio Inc.) were used for the detection of VEGF cDNA.

Western blot analysis was performed using antibodies that specifically recognize proteins, including eNOS (total-eNOS; Cell Signaling Technology, MA, USA) and phospho-eNOS at Ser1177 (p-eNOS; Cell Signaling Technology). The regions containing p-eNOS and eNOS proteins were visualized by electrochemiluminescence Western blotting luminal reagent (Santa Cruz Biotechnology, Dallas, TX). The extent of p-eNOS was normalized by that of eNOS.

### Porcine Model of Chronic Myocardial Ischemia

**Figure 2:**
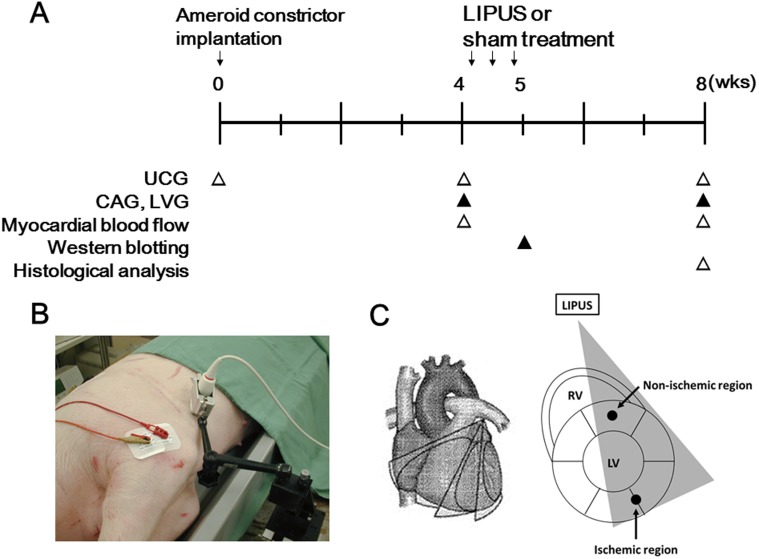
Experimental protocol. (**A**) Protocol, (**B**) LIPUS therapy in action, and (**C**) schematic images of LIPUS application. UCG, ultrasound cardiography; CAG, coronary angiography; LVG, left ventriculography.

We used a total of 60 domestic pigs (25 to 30 kg in body weight) in the present study. After sedation with medetomidine (0.05 mg/kg, IM) and midazolam (0.3 mg/kg, IM), we maintained the anesthesia with an inhalation of 2–5% sevoflurane for implantation of an ameroid constrictor, LIPUS treatment, and euthanization by intravenous injection of 5% potassium chloride at 8 weeks after implantation of an ameroid constrictor (**Figure 2**). We opened the chest, suspended the pericardium and left atrial appendage, revealed the left circumflex coronary artery (LCx), and put an ameroid constrictor (Research Instruments NW, Lebanon, OR) around the proximal LCx to gradually induce a total occlusion of the artery in 4 weeks without causing myocardial infarction [4]. This model is widely used to examine the effect of an angiogenic therapy on the ischemic hibernating myocardium [4], [13], [14]. In the present study, animals that were suspected of developing myocardial infarction (e.g. severe segmental LV wall thinning) or showed left ventricular ejection fraction (LVEF) of more than 55% based on echocardiographic studies at 4 weeks were excluded before randomization. Finally, 28 pigs were assigned to the 2 groups with (LIPUS group) or without (control group) the LIPUS therapy using block randomization with a block size of 6. We defined absolute change in LVEF at 8 weeks between the control and the LIPUS group as the primary end-point, with an unpaired *t* test and α = 5%. Assuming that the LIPUS therapy exerts similar effects as in our prior SW therapy, the absolute improvement of 10% in LVEF at 8 weeks was the assumed effect size for our sample size calculation. Power analysis was conducted in R version 3.0.3. A total of 8 pigs ensured a statistical power of 90% based on our assumption. Thus, the present study (n = 9 each) has a sufficient statistical power.

### LIPUS Therapy to Chronic Ischemic Myocardium

On the basis of the in vitro experiment, LIPUS (20-min irradiation at 32-cycle) was applied to the lateral wall (ischemic region) by 2-dimensional scan at 3 different short-axis levels, including (1) basal LV, (2) papillary muscle level, and (3) near the apex of a heart (**Figure 2**). Among the 28 pigs used, we treated 14 pigs with the LIPUS treatment at 4 weeks after the implantation of an ameroid constrictor (LIPUS group, 3 times a week), while we treated the remaining 14 pigs with the same anesthesia procedures for 70 min but without the LIPUS treatment (control group, 3 times a week).

### Coronary Angiography and Left Ventriculography

After systemic heparinization (30,000 U/body IV), we performed coronary angiography and left ventriculography in a left oblique view with the use of a cineangiography system (Toshiba Medical Systems, Ohtawara, Japan) (n = 9 each) [4]. We semi-quantitatively evaluated the extent of collateral flow to the LCx region by the Rentrop score (0, no visible collateral vessels; 1, faint filling of side branches of the main epicardial vessel without filling the main vessel; 2, partial filling of the main epicardial vessel; 3, complete filling of the main vessel) [15]. We also counted the number of visible coronary arteries in the LCx region. To compare the extent of collateral development at a given time, we selected the frame in which the whole left anterior descending coronary artery (LAD) was first visualized [4].

### Echocardiographic Evaluation

We performed transthoracic echocardiographic studies before ameroid implantation (baseline) and at 4 and 8 weeks after the procedure (n = 9 each). To evaluate regional myocardial function, we calculated wall thickening fraction (WTF) by using the following formula; WTF = 100×(end-systolic wall thickness − end-diastolic wall thickness)/end-diastolic wall thickness [4].

### Measurement of Regional Myocardial Blood Flow

We evaluated regional myocardial blood flow (RMBF) with colored microspheres (Dye-Trak; Triton Technology, San Diego, CA) at 4 and 8 weeks after implantation (n = 7 each) [4]. Due to technical problems, 2 samples in each group were excluded. We injected microspheres through the left atrium and aspirated a reference arterial blood sample from the descending aorta at a constant rate of 20 ml/min for 60 seconds using a withdrawal pump. We extracted microspheres from the left ventricular (LV) wall and blood samples by potassium hydroxide digestion, extracted the dyes from the spheres with dimethylformamide (200 µl), and determined their concentrations by spectrophotometry [16]. We calculated myocardial blood flow (ml · min^−1^ · g^−1^) of the LCx region and the IVS region [4].

### Factor VIII Staining

We treated paraffin-embedded sections with a rabbit anti-factor VIII antibody (N1505; Dako, Copenhagen, Denmark). We counted the number of factor VIII-positive cells in 10 fields of the ischemic LV lateral wall (LCx region) and the non-ischemic interventricular septum (IVS) (LAD region) in each heart at x400 magnification (n = 7 each) [4].

### Western Blot Analysis in Vivo

The sections from the ischemic LCx region and the non-ischemic IVS region were used for Western blot analysis (n = 5 each). The regions containing VEGF, eNOS, and bFGF proteins were visualized by electrochemiluminescence Western blotting luminal reagent (Santa Cruz Biotechnology). The extent of the VEGF, eNOS, and bFGF was normalized by that of glyceraldehyde-3-phosphate dehydrogenase (GAPDH) [4].

### Statistical Analysis

Results are expressed as mean ± SEM. Comparisons of parameters between 2 groups were performed with unpaired Student’s t-test. Data were analyzed by one-way ANOVA (in vitro study) or two-way ANOVA (in vitvo study) followed by Tukey’s HSD test for multiple comparisons. A value of P*<*0.05 was considered to be statistically significant.

## Results

### Acoustic Pressure of LIPUS

When the number of cycles was increased from 1 to 16, 32, 48 and 64, peak acoustic pressure generated by a 1.875 MHz transducer was decreased from 1.59 to 0.77, 0.56, 0.43 and 0.36 MPa, respectively (**Table S1**). The relationships between voltage applied to each transducer element and acoustic pressure are shown in **Figure S1**. Among the conditions tested, estimated Ispta was highest when the cycle number was 32 (**Table S1**).

### Effects of LIPUS on mRNA Expression of VEGF, Flk-1 and bFGF, and eNOS Activity in HUVECs

In accordance with the preliminary data mentioned above (**Table S1**), the LIPUS treatment significantly up-regulated mRNA expression of VEGF in HUVECs, with a maximum effect noted at 32 cycles (1.33-fold increase, P<0.05) (**Figure 1**) while the expression of its receptor Flk-1 was not enhanced (Control, 1.00±0.06 vs. LIPUS, 1.01±0.10; at 32 cycles). Although the LIPUS therapy at 32 cycles did not up-regulated mRNA expression of bFGF, the ratio of phospho-eNOS to total-eNOS (p-eNOS/total-eNOS), a marker of eNOS activation, was significantly increased in HUVECs (1.43-fold increase, P<0.05) (**Figure S2**).

### Effects of the LIPUS Therapy on Ischemia-Induced Myocardial Dysfunction

**Figure 3:**
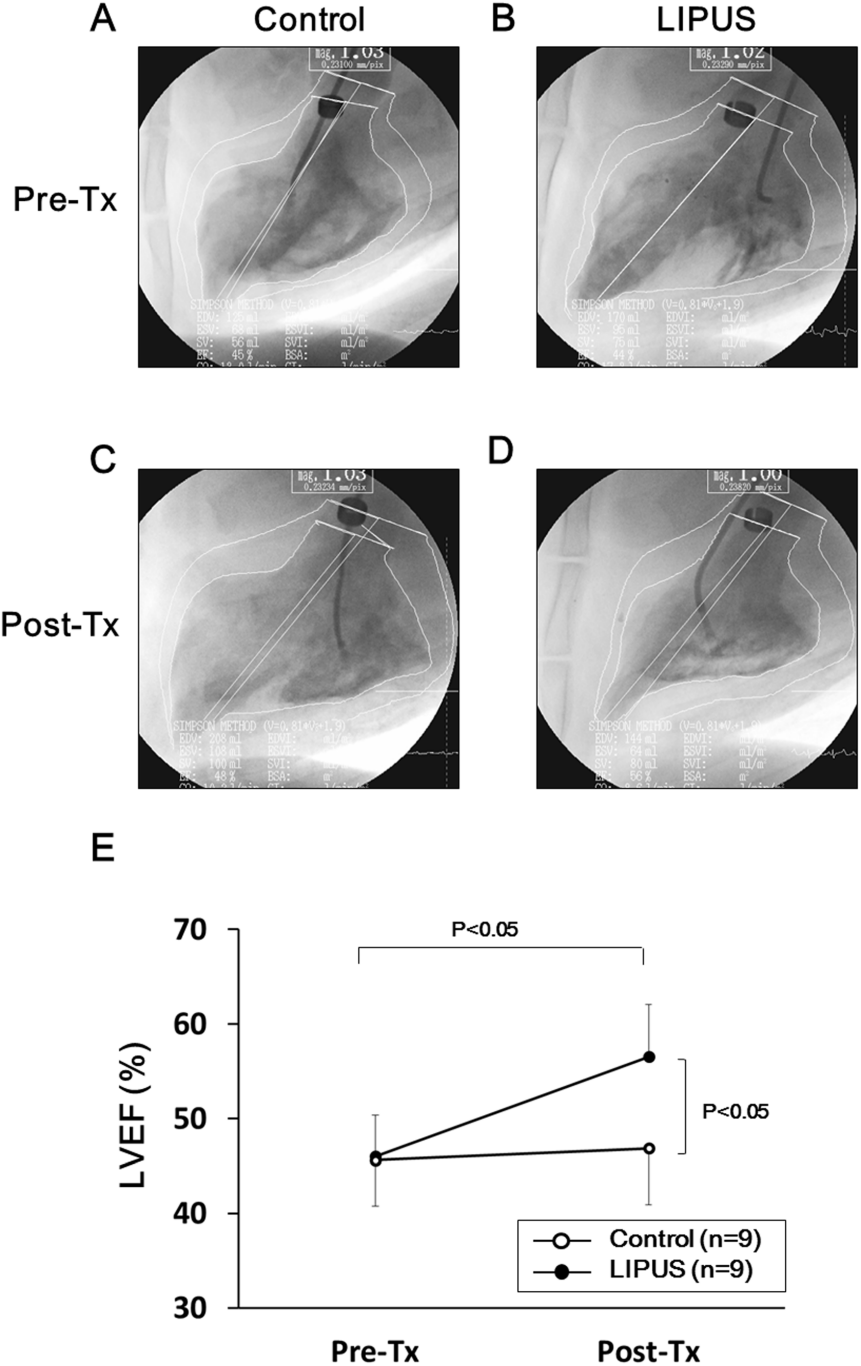
LIPUS therapy improved ischemia-induced myocardial dysfunction in vivo. (**A**, **B**) Four weeks after the implantation of an ameroid constrictor (pre-Tx), LV wall motion of the LCx region was reduced in both the control (**A**) and the LIPUS group (**B**). (**C**, **D**) Four weeks after the first left ventriculography (post-Tx), no significant change in LV wall motion was noted in the control group (**C**), whereas marked recovery was noted in the LIPUS group (**D**). **E**, The LIPUS therapy normalized left ventricular ejection fraction in the LIPUS group but not in the control group. Results are expressed as mean ± SEM (n = 9 each).

**Figure 4:**
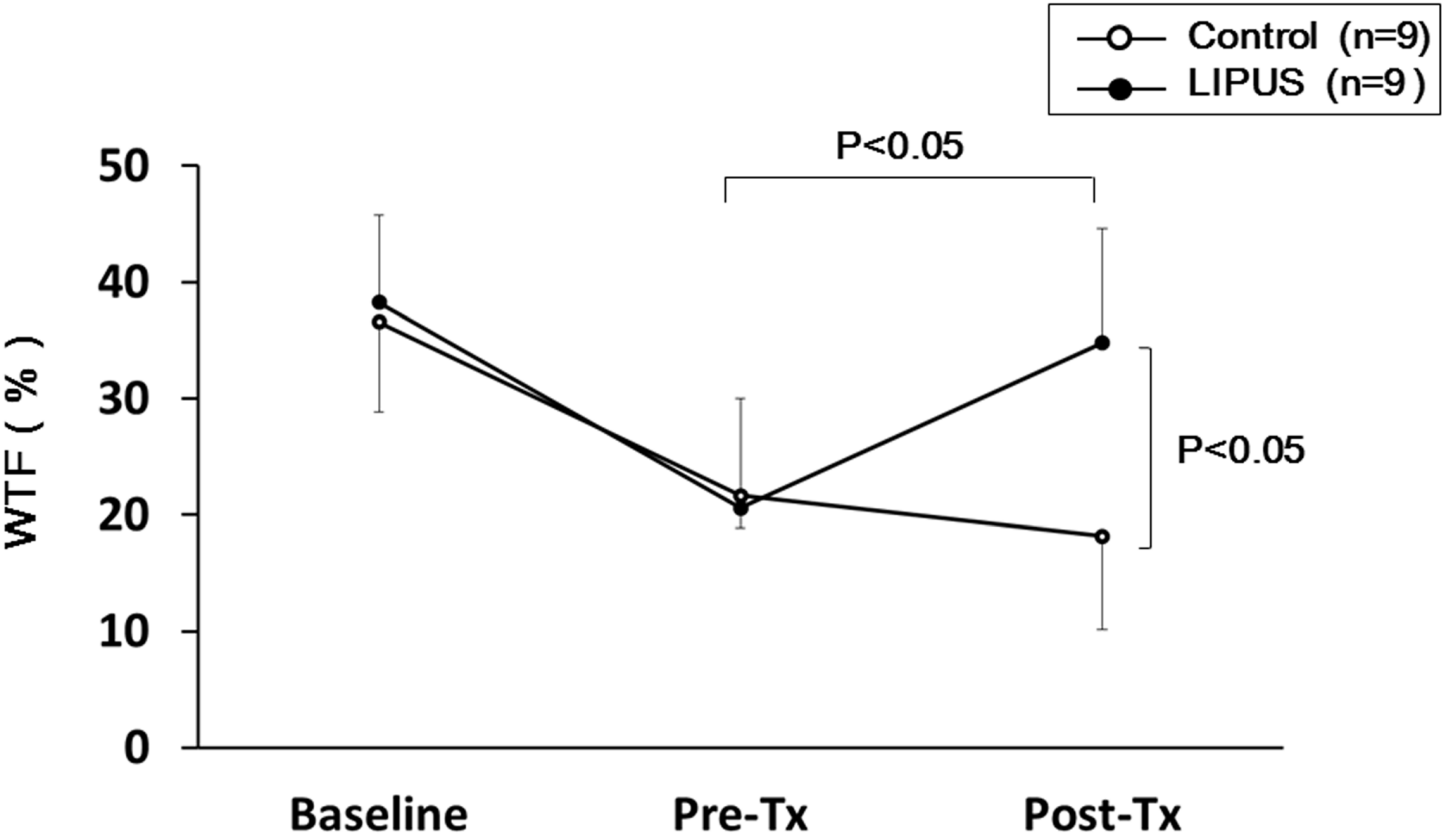
LIPUS therapy improved regional myocardial function in vivo. LIPUS therapy induced a complete recovery of WTF of the ischemic lateral wall. Results are expressed as mean ± SEM (n = 9 each).

At 4 weeks after ameroid implantation (Pre-Tx), left ventriculography demonstrated reduced LVEF in both groups (**Figure 3A and 3B**). At 8 weeks (Post-Tx), while LVEF remained unchanged in the control group (45.6±4.6 to 46.9±5.6%, P = 0.33) (**Figure 3C**), it was normalized in the LIPUS group (46.0±4.1 to 56.6±5.2%, P<0.05) (**Figure 3D**). Absolute improvement of LVEF 4 weeks after the therapy, the primary end-point of the present study, was significantly greater in the LIPUS than in the control group (46.9±5.6 to 56.6±5.2%, P<0.05) (**Figure 3E**). We also serially measured WTF of the LCx region (lateral wall of the LV) by transthoracic echocardiography. While WTF was equally reduced at 4 weeks in both groups (Control, 21.6±2.7 vs. LIPUS, 20.6±9.4%), WTF was markedly improved in the LIPUS group, but remained unchanged in the control group at 8 weeks (Control, 18.2±8.0 vs. LIPUS, 34.8±9.8%, P*<*0.05) (**Figure 4**). Detailed data of left ventriculography and echocardiography are shown in **Table S2**.

### Angiogenic Effects of the LIPUS Therapy

**Figure 5:**
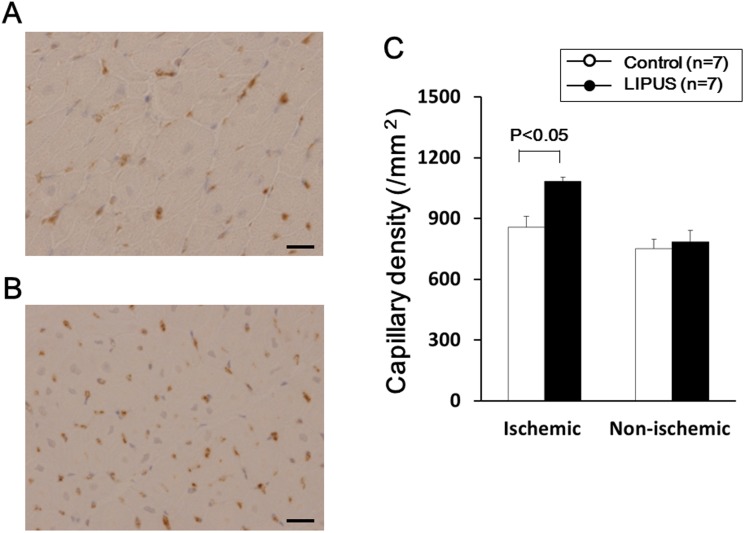
LIPUS therapy increased the density of factor VIII-positive capillaries in the ischemic myocardium. (**A, B**) Factor VIII staining of the LCx region from the control (**A**) and the LIPUS group (**B**). Scale bar represents 20 µm. **C.** Capillary density was significantly higher in the LIPUS group than in the control group in the ischemic lateral wall. Results are expressed as mean ± SEM (n = 7 each).

There was no difference in the coronary artery dominance between the 2 groups; in the control group, right dominant in 0, balanced type in 7, left dominant in 2 and in the LIPUS group, right dominant in 0, balanced type in 7, and left dominant in 2 [17]. At 4 weeks after ameroid implantation, coronary angiography demonstrated a total occlusion of the LCx, which was perfused via collateral vessels with severe delay in both the control (**Figure S3A**) and the LIPUS groups (**Figure S3B**). However, at 8 weeks, the LIPUS group **(Figure S3D**), but not the control group (**Figure S3C**), showed a marked development of coronary collateral vessels in the ischemic LCx region, with an increased Rentrop score (**Figure S3E**) and an increased number of visible coronary arteries in the region (**Figure S3F**). Factor VIII staining showed that the number of factor VIII-positive capillaries was increased only in the LIPUS group (**Figure 5A and 5B**). Quantitative analysis demonstrated that the number of capillaries in the ischemic lateral wall region at 8 weeks was significantly higher in the LIPUS group than in the control group (Control, 858±151 vs. LIPUS, 1084±175/mm^2^, P*<*0.05), whereas it was unchanged in the non-ischemic IVS region in both groups (Control, 751±20 vs. LIPUS, 785±45/mm^2^, P* = *0.27) (**Figure 5C**).

### Effects of the LIPUS Therapy on Regional Myocardial Blood Flow

**Figure 6:**
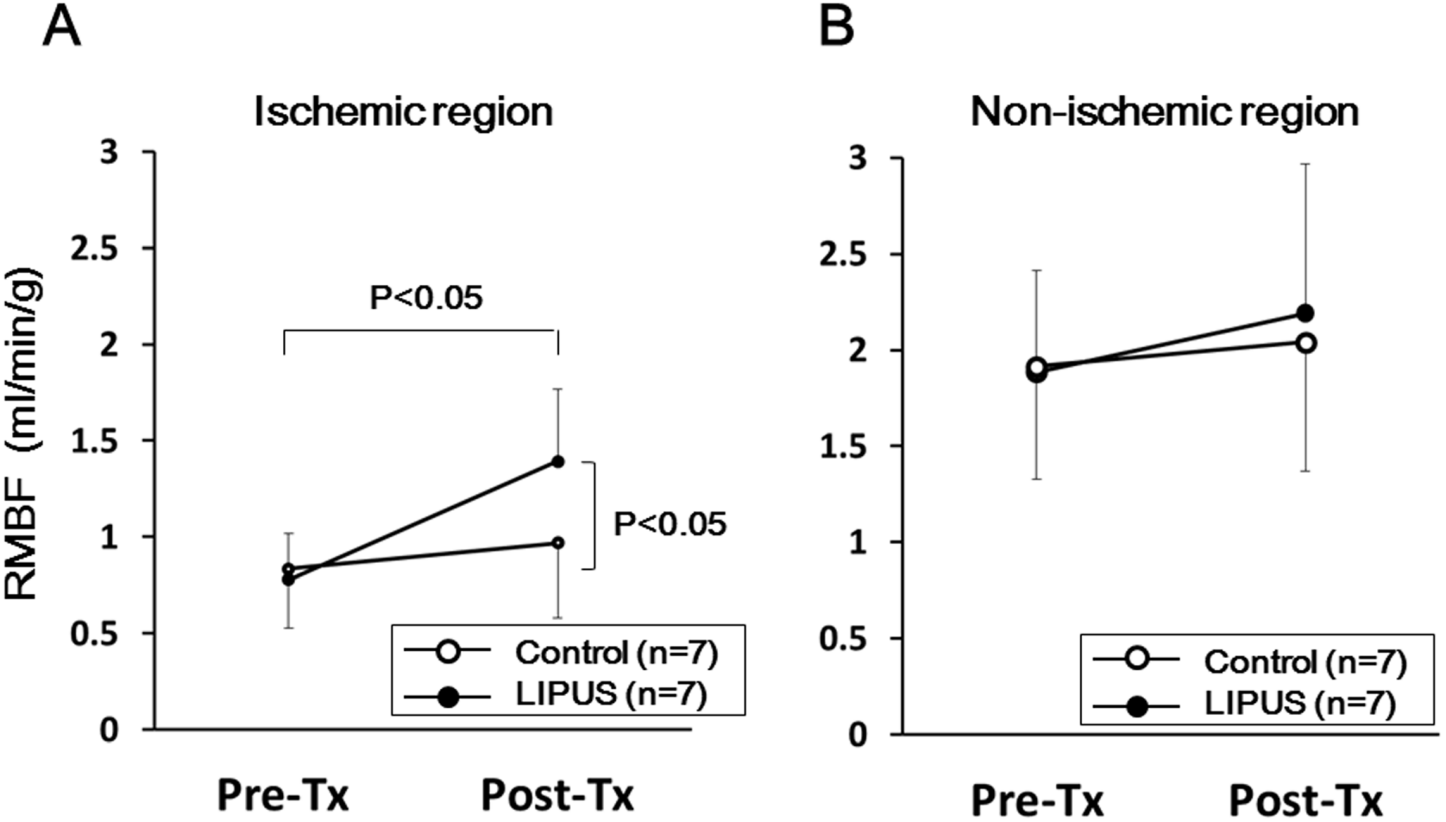
LIPUS therapy improved RMBF in the ischemic region in vivo. LIPUS therapy significantly increased RMBF, assessed by colored microspheres in the lateral wall (**A**), while it was not changed in the both groups in the non-ischemic IVS region (**B**). Results are expressed as mean ± SEM (n = 7 each).

At 4 weeks, RMBF was equally decreased in the lateral wall in both groups (Control, 0.84±0.29 vs. LIPUS, 0.78±0.22 ml/min/g, P* = *0.35), but remained normal in the IVS (Control, 1.91±0.59 vs. LIPUS, 1.89±0.53 ml/min/g, P* = *0.47). At 8 weeks, the LIPUS treatment significantly improved RMBF in the lateral wall (Control, 0.97±0.36 vs. LIPUS, 1.39±0.35 ml/min/g, P*<*0.05) (**Figure 6A**), whereas it remained unchanged in the non-ischemic IVS in both groups despite the LIPUS treatment (Control, 2.04±0.69 vs. LIPUS, 2.19±0.77 ml/min/g, P* = *0.36) (**Figure 6B** **)**.

### Effects of the LIPUS Therapy on Expression of VEGF, eNOS and bFGF

**Figure 7:**
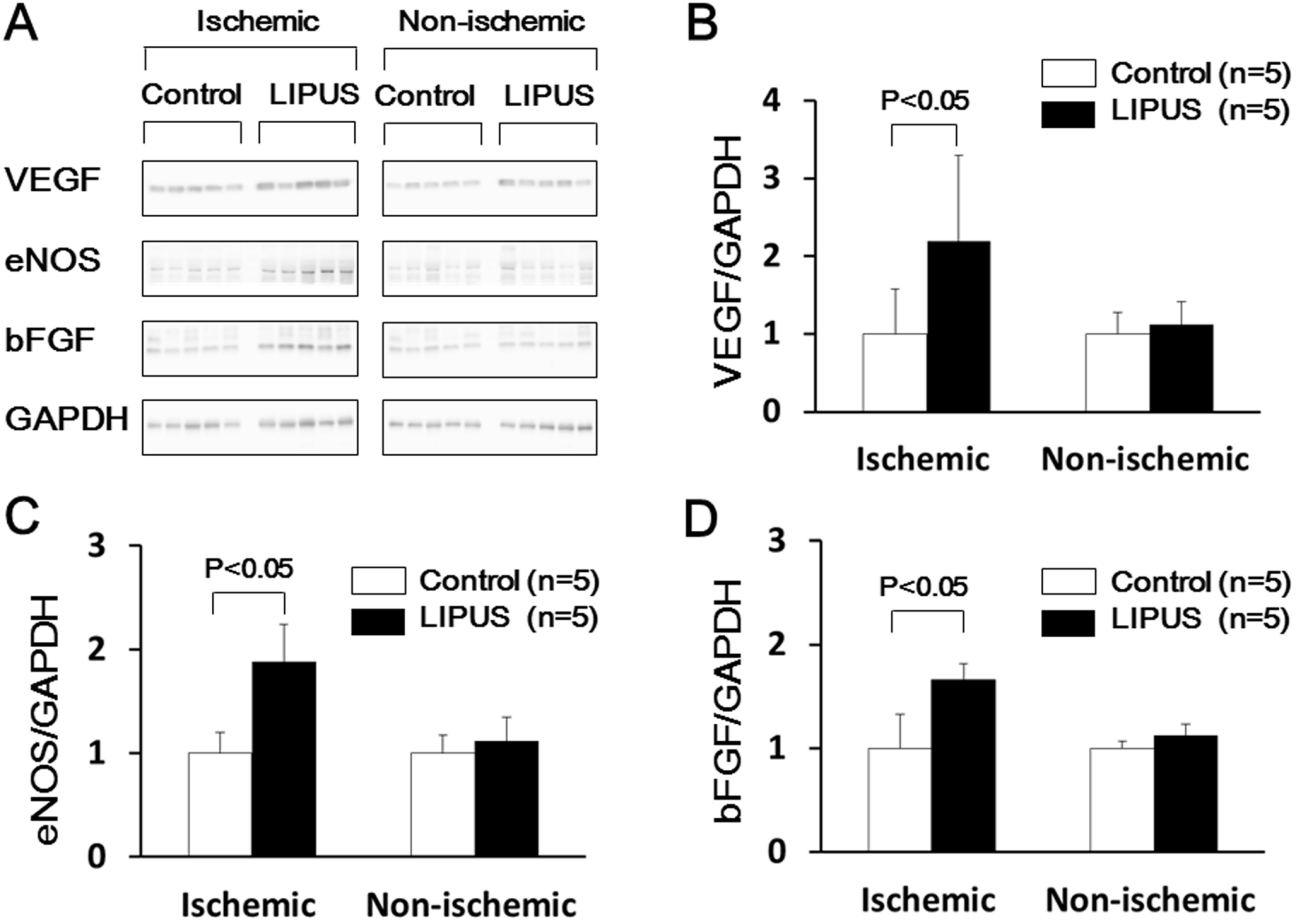
LIPUS treatment up-regulated protein expression of VEGF, eNOS, and bFGF in the ischemic myocardium (n = 5 each). (A) Western blots of VEGF, eNOS, and bFGF. (B∼D) Quantification of VEGF, eNOS, and bFGF expression.

Western blot analysis at 5 weeks demonstrated that protein expressions of VEGF, eNOS and bFGF were significantly up-regulated in the lateral wall in the LIPUS group, whereas they were unchanged in the non-ischemic IVS region in both groups despite the LIPUS treatment (**Figure 7**).

### Safety of the LIPUS Therapy

No adverse hemodynamic changes or no histological damage in the skin or lung were noted by the LIPUS.

## Discussion

The novel finding of this study is that the LIPUS therapy induces angiogenesis in the ischemic myocardium and normalizes myocardial function in a porcine model of chronic myocardial ischemia in vivo. To the best of our knowledge, this is the first report that demonstrates the potential usefulness of the LIPUS therapy as a non-invasive treatment of IHD.

### Enhancing Effect of the LIPUS on Endothelial Regeneration in Vitro

The number of cycles of pulsed ultrasound of our equipment in diagnostic use is 1 cycle while we tested larger numbers of cycles (16, 32, 48 and 64) in the present study for therapeutic purpose. When the LIPUS was applied to HUVECs, the maximal effect to up-regulate mRNA expression of VEGF was achieved at 32 cycles. The peak acoustic pressure was reduced with increasing number of cycles while elevation of voltage applied to each transducer element was limited by temperature rise of the ultrasound probe although higher the voltage could induce higher peak acoustic pressure. As a result, the highest estimated Ispta was obtained when the LIPUS was applied at 32 cycles in the present study. The intensity of the ultrasound used in the present study is equivalent to that of ultrasound diagnostic devices while higher intensity ultrasound is used for thrombolysis or tumor ablation (high intensity focused ultrasound, HIFU) [9]. Although we confirmed the beneficial effect of the LIPUS at 32 cycles in vivo with enhanced angiogenesis and improved LV myocardial dysfunction, it remains to be further examined which was more important, higher Ispta or the cycle number of 32 itself. This point needs to be examined in future studies.

### Beneficial Effects of the LIPUS Therapy on IHD

In the present study, we demonstrated that the LIPUS therapy (1) normalized global and regional myocardial function in vivo, (2) increased capillary density and RMBF of the chronically ischemic region without any adverse effects, and (3) enhanced protein levels of VEGF, eNOS, and bFGF in the ischemic myocardium without affecting those in the non-ischemic myocardium. These results indicate that the LIPUS therapy is an effective and safe therapeutic strategy for ischemia-induced myocardial dysfunction.

### Potential Mechanisms for LIPUS-induced Angiogenesis

We demonstrated that the LIPUS up-regulated VEGF expression and eNOS activity in vitro and enhanced angiogenesis in association with an enhanced protein expression of VEGF, eNOS, and bFGF in pigs in vivo. Barzelai et al. also reported that LIPUS induces angiogenesis and up-regulation of VEGF in hind-limb ischemia in rats [10]. eNOS has vasodilating and angiogenic effects [18], while bFGF is known to mediate new blood vessel formation in wound healing [19]. Thus, it is suggested that the LIPUS therapy improved myocardial function by enhancing multiple angiogenic pathways. However, it is unclear how LIPUS regulates the expression of angiogenic factors in the ischemic myocardium alone.

In the present study, the extent of an increase in VEGF mRNA expression by a single LIPUS therapy for 20 min was rather small in HUVECs in vitro, whereas the protein expression of VEGF increased by more than twice after the LIPUS therapy (3 times a week) in vivo (2.19-fold increase, P<0.05; **Figure 7B**). Also, the LIPUS therapy did not up-regulate the mRNA expression of bFGF in vitro, whereas the LIPUS therapy significantly enhanced the protein expression of bFGF in vivo. These results suggest that repeated therapies up-regulate angiogenic factors more than single therapy. Another possibility is that cell-to-cell interaction between endothelial cells and other types of cells is important in enhancing multiple angiogenic pathways [20]. Bone marrow-derived progenitor cells and residential stem cells have been shown to contribute to angiogenesis process [21]. Xu et al. reported that LIPUS stimulated hematopoietic stem/progenitor cell viability, proliferation and differentiation in vitro [22]. Toyama et al. also reported that application of LIPUS to circulating angiogenic cells augmented their generation and migration capacities [23]. Thus, it is possible that the LIPUS directly and/or indirectly affects the function and dynamics of immature cells, such as bone marrow-derived mononuclear cells and residential cardiac stem cells [24].

### Other Potential Mechanisms of LIPUS-induced Beneficial Effects on LV dysfunction

Previous studies have shown that LIPUS stimulates the production of angiogenic cytokines (e.g. IL-8, bFGF, and VEGF) [25] and reduces COX-2 and PGI_2_ production [26], and that the low-energy SW therapy exerts multiple effects, including angiogenic, anti-inflammatory, anti-oxidant and anti-apoptotic effects [4]–[8], [27]. Thus, as in the case with the low-energy SW therapy, the mechanisms other than angiogenesis may also contribute to the beneficial effects of LIPUS in the present study. This point remains to be examined in future studies.

### The Effects of LIPUS on the Single-cell Level

Mechanical stimuli are known to affect endothelial cell function and mechanosensors on endothelial cell membranes, such as integrins and caveolins [28], [29]. Ultrasound irradiation has been reported to produce shear stress on endothelial cells [11]. Also, mechanical stimulation on cultured endothelial cells induced by shear stress has been reported to enhance endothelial cell proliferation, migration and sprouting [30]. In addition, ultrasound has been shown to induce sonoporation on endothelial cell membrane leading to influx of calcium ion [31]. Thus, it is conceivable that several mechanisms, such as triggering of mechanosensors, activation of endothelial cell growth and sonoporation-activated alterations, contribute to the LIPUS-induced angiogenesis. Further studies are needed.

### Comparison of SW Therapy and LIPUS Therapy

We have previously demonstrated that low-energy cardiac SW therapy effectively induces neovascularization and improves myocardial function in a porcine model of chronic myocardial ischemia [4]. Based on the promising results in animal studies, we have developed the low-energy SW therapy, where the low-energy SW therapy improves symptoms and myocardial perfusion in patients with severe angina pectoris [5]–[8]. We also have demonstrated that the low-energy SW therapy improves the walking ability of patients with peripheral artery disease (PAD) and intermittent claudication [32]. Furthermore, we have recently demonstrated that the low-energy SW therapy induces therapeutic lymphangiogenesis, enhances skin wound healing and improves locomotor recovery after spinal cord injury [33]–[35]. Since SW and ultrasound share similarities as they similarly travel straight through body tissue (fat, muscles, body fluid etc.), we tested potential usefulness of LIPUS for the treatment of ischemic heart disease as a non-invasive angiogenic therapy. In the present study, we were able to demonstrate that the LIPUS at a certain irradiation condition effectively enhances the expression of angiogenic cytokines and significantly improves ischemia-induced myocardial dysfunction in vivo. Since the LIPUS therapy on ischemic myocardium was equally effective to the low-energy SW therapy [4], the LIPUS therapy and the low-energy SW therapy may activate common signaling pathways.

The intensity of the ultrasound used in the present study is within the limits of acoustic output standards for diagnostic ultrasound devices defined by U.S. Food and Drug Administration. Since the present LIPUS system is characterized by the use of the same probe for both diagnostic purpose and for LIPUS therapy, which makes the LIPUS therapy device much easier to handle than the SW therapy device, and thus, the LIPUS therapy is expected as a new, non-invasive angiogenic therapy because ultrasound diagnostic devices have already been widely used in the world.

### Clinical Implications

As mentioned above, the LIPUS therapy is expected to be effective in patients with IHD. Unlike SW that is applied to focused region, LIPUS is irradiated not only to ischemic region but also to non-ischemic region. However, the LIPUS therapy enhanced angiogenesis only in the ischemic region without adverse effects. We consider that this new LIPUS therapy should be first applied for patients with symptomatic angina pectoris without indication for PCI or CABG. The LIPUS therapy is also expected to be effective in PAD patients with intermittent claudication or refractory skin ulcer like the low-energy SW therapy [32]–[34].

### Study Limitations

Several limitations should be mentioned for the present study. First, we optimized the setting of ultrasound within the intensity of acoustic output standards for diagnostic devices because higher intensity of ultrasound involves temperature rise in the probe which may cause burn injury. To give priority to safety, we did not examine higher intensity of ultrasound to prevent skin burn due to generation of heat. Second, in the present study, we performed the LIPUS therapy 3 times a week based on our previous studies with cardiac SW therapy [4], [5], [7], [36], [37] and observed beneficial effects of the LIPUS therapy on ischemia-induced LV dysfunction. However, it is still not clear whether the 3 times is the optimal number of therapies or not. This point remains to be addressed in future studies.

### Conclusions

In the present study, we were able to demonstrate that the non-invasive LIPUS therapy enhances multiple angiogenic pathways, effectively increases capillary density and regional myocardial blood flow (RMBF), and normalizes ischemia-induced myocardial dysfunction without any adverse effects. Thus, these results indicate that the LIPUS therapy may be an effective, safe, and non-invasive therapy for IHD.

## Supporting Information

Figure S1 The relationship between voltage applied to each transducer element and acoustic pressure at various cycle numbers. The each circle represents the highest acoustic pressure achieved at each cycle number. Elevation of voltage applied to each transducer element was limited by temperature rise of the ultrasound probe.
(TIF)

Figure S2 In vitro study. (**A**) LIPUS therapy did not up-regulated mRNA expression of eNOS or bFGF at 32-cycle. Results are expressed as mean ± SEM (n = 8 each). (**B**) LIPUS therapy significantly increased the ratio of phospho-eNOS to total-eNOS (p-eNOS/total-eNOS), a marker of eNOS activation at 32-cycle in vitro. Results are expressed as mean ± SEM (n = 6 each).
(TIF)

Figure S3 LIPUS therapy enhanced coronary angiogenesis in vivo. (**A**, **B**) Four weeks after the implantation of an ameroid constrictor (pre-Tx) LCx was totally occluded and was perfused via collateral vessels with severe delay in both the control group (**A**) and the LIPUS group (**B**). (**C**, **D**) Four weeks after the first coronary angiography (post-Tx), no significant change in coronary vessels was noted in the control group (**C**), whereas a marked development of visible coronary vessels was noted in the LIPUS group (**D**). (**E**, **F**) Four weeks after the first coronary angiography, no significant increase in the Rentrop score (**E**) or visible coronary arteries from LCx (**F**) was noted in the control group, whereas increased Rentrop score and a marked development of visible coronary vessels were noted in the LIPUS group. Results are expressed as mean ± SEM (n = 9 each).
(TIF)

Table S1 **The relationships between voltage and acoustic pressure.**
(DOC)

Table S2 **Findings of left ventriculography and echocardiography.**
(DOC)
