# Supplementary figures and images for: Low-Intensity Pulsed Ultrasound Induces Angiogenesis and Ameliorates Left Ventricular Dysfunction in a Porcine Model of Chronic Myocardial Ischemia
Source: PLoS One. 2014 Aug 11;9(8):e104863. doi: 10.1371/journal.pone.0104863 (PMC4128732; doi:10.1371/journal.pone.0104863)

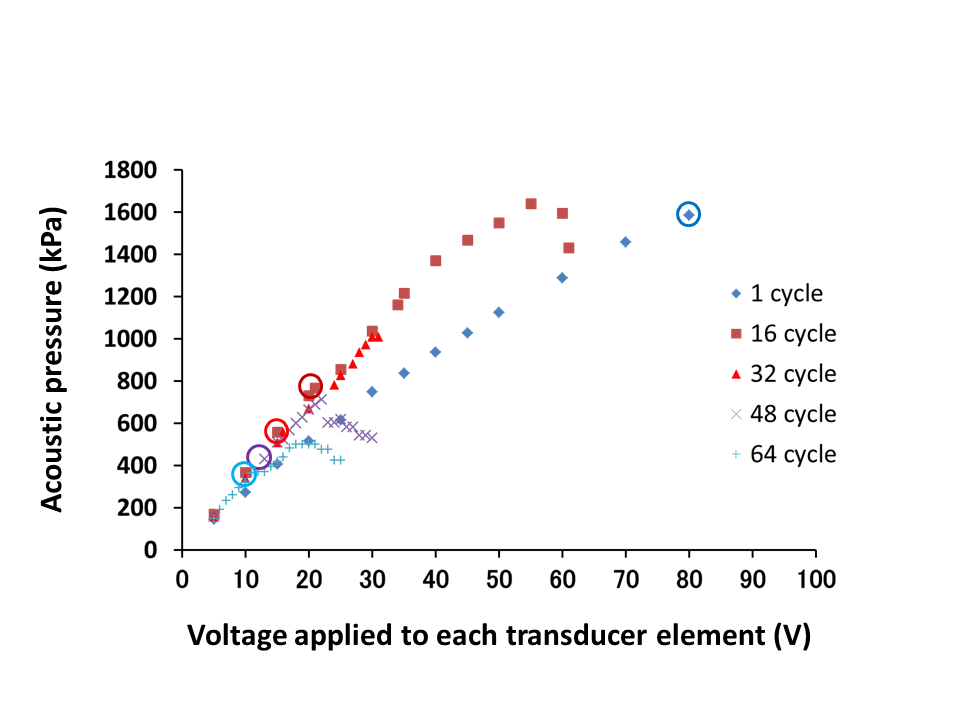

Supplement: Figure S1 — The relationship between voltage applied to each transducer element and acoustic pressure at various cycle numbers. The each circle represents the highest acoustic pressure achieved at each cycle number. Elevation of voltage applied to each transducer element was limited by temperature rise of the ultrasound probe. (TIF) [file pone.0104863.s001.tif]
