# Supplementary material for: Low-Intensity Pulsed Ultrasound Induces Angiogenesis and Ameliorates Left Ventricular Dysfunction in a Porcine Model of Chronic Myocardial Ischemia
Source: PLoS One. 2014 Aug 11;9(8):e104863. doi: 10.1371/journal.pone.0104863 (PMC4128732; doi:10.1371/journal.pone.0104863)
Supplement: Table S1 — The relationships between voltage and acoustic pressure. (DOC) [file pone.0104863.s004.doc]

**Table S1. The relationships between voltage and acoustic pressure.**

|  | Voltage (V) | Acoustic pressure  (kPa) | Estimated Ispta  (mW/cm^2^) | Estimated Ispta  (Relative to 1 cycle) |
| --- | --- | --- | --- | --- |
| 1 cycle | 88.1 | 1.59×10^3^ | 166 | 1 |
| 16 cycle | 20.4 | 0.77×10^3^ | 151 | 0.91 |
| 32 cycle | 16.3 | 0.56×10^3^ | 193 | 1.16 |
| 48 cycle | 13.1 | 0.43×10^3^ | 188 | 1.13 |
| 64 cycle | 11.3 | 0.36×10^3^ | 186 | 1.12 |

Ispta, spatial peak temporal average intensity.
