# Supplementary material for: Low-Intensity Pulsed Ultrasound Induces Angiogenesis and Ameliorates Left Ventricular Dysfunction in a Porcine Model of Chronic Myocardial Ischemia
Source: PLoS One. 2014 Aug 11;9(8):e104863. doi: 10.1371/journal.pone.0104863 (PMC4128732; doi:10.1371/journal.pone.0104863)
Supplement: Table S2 — Findings of left ventriculography and echocardiography. (DOC) [file pone.0104863.s005.doc]

**Table S2. Findings of left ventriculography and echocardiography.**

|  | Control 　　　　Pre-Tx | | Control 　Post-Tx | | | LIPUS Pre-Tx | | LIPUS Post-Tx |
| --- | --- | --- | --- | --- | --- | --- | --- | --- |
| Body weight (kg) | | 49.0±1.1 | | 69.1±1.1 * | 48.4±1.2 | | 67.3±1.7 * | |
| LVG  LVEDV (ml)  LVESV (ml)  LVEF (%) UCG  LVDd (mm)  LVDs (mm)   LVEF (%)  WTF (%) | | 137±18.4 74±9.5 45.6±4.6  44.3±1.6 31.4±1.1 49.6±1.1 21.6±2.7 | | 169±11.8 *  89±7.1 46.9±5.6  47.9±0.8 33.3±0.6 51.3±1.1 20.6±9.4 | 140±9.7 75±5.7 46.0±4.1  43.9±1.2 31.4±0.9 48.6±1.2 18.2±8.0 | | 154±8.5 * 67±2.8 *^, †^ 56.6±5.2 *^, †^  46.8±1.1 30.2±0.6 ^†^ 58.2±1.2 *^, †^ 34.8±9.8 *^, †^ | |

LVG; left ventriculography. UCG; ultrasound echocardiography.

*P<0.05; Pre-Tx vs. Post-Tx. †P<0.05; control group vs. LIPUS group.
